# Focal Choroidal Excavation: Epidemiology, Clinical Characteristics and Multimodal Imaging Findings

**DOI:** 10.3390/diagnostics13040602

**Published:** 2023-02-07

**Authors:** Paulina Szabelska, Justyna Mędrzycka, Joanna Brydak-Godowska, Radosław Różycki, Joanna Gołębiewska

**Affiliations:** 1 Department of Ophthalmology, Military Institute of Aviation Medicine, 01-755 Warsaw, Poland; 2 Department of Ophthalmology, Infant Jesus Clinical Hospital, University Clinical Center, Medical University of Warsaw, 02-091 Warsaw, Poland

**Keywords:** focal choroidal excavation (FCE), pachychoroid, pachychoroid spectrum diseases, optical coherence tomography (OCT), OCT Angiography (OCTA)

## Abstract

Background: Focal choroidal excavation (FCE) is one of the pachychoroid spectrum diseases. It may be an isolated lesion or associated with other ophthalmological disorders. The aim of the study was to present the epidemiology, clinical features and multimodal imaging findings in FCE. Methods: This is a case series of 14 consecutive patients with a diagnosis of FCE, confirmed by multimodal imaging, from a review of the 5076 optical coherence tomography (OCT) scans in 2538 patients. Choroidal thickness (CT) was measured under the fovea and in the area of maximum choroidal thickening in the affected eye and under the fovea in the fellow eye. Results: The mean age of the subjects was 40 ± 13.58 years. FCE occurred unilaterally and was an isolated lesion in all cases. The fellow eye did not show any macular pathology in all patients. Twelve eyes presented conforming FCEs and two non–conforming FCEs. In 79% of cases, FCE was subfoveal. The mean maximum CT was 390 μm in the affected eye with the presence of pachyvessels. A total of 13 patients were asymptomatic, while one patient reported a visual disturbance due to neovascularization secondary to FCE. Of all the multimodal imaging techniques, optical coherence tomography (OCT) provided the most important data in the diagnosis of FCE. Conclusions: Our study confirmed that FCE is a rare ocular condition, but it may be more common in Caucasian population than previously known. Multimodal imaging methods, mainly OCT, are crucial in FCE diagnostics. Further studies are needed to expand the available knowledge about its etiology and clinical course.

## 1. Introduction

In 2006, Jampol et al. were the first to describe a unilateral localized choroidal depression with near-normal overlying retinal architecture and good visual acuity in a 62-year-old myopic woman, using time domain optical coherence tomography (TD–OCT) [1]. In 2010, Wakabayashi et al. reported on three Asian women with unilateral choroidal excavation using spectral domain optical coherence tomography (SD–OCT), which allowed more detailed visualization of the retinal and choroidal structure than TD–OCT [2]. The excavation involved the outer retinal layers up to the external limiting membrane (ELM) in two cases and only retinal pigment epithelium (RPE) in one eye, with a normal foveal contour and intact inner retinal layers. 

Focal choroidal excavation (FCE) was firstly used as a term by Margolis et al. in 2011 [3], who described this pathology in 12 patients, including one with bilateral lesions. FCE is one of the pachychoroid spectrum diseases, a group of entities that share common phenotypic features—increased choroidal thickness (CT) (above 300 μm) and dilatation of the Haller’s layer vessels (called pachyvessels) which compress the overlying choriocapillaris and Sattler’s layer, with or without concomitant macular neovascularization (MNV) [4]. The choroidal thickening may be focal or diffuse, foveal or extrafoveal. In the case of extrafoveal thickening, CT at this area may be at least 50 μm greater than subfoveally [4]. 

According to the available literature, the etiology of FCE remains unclear. It is supposed that FCE may be a congenital posterior segment malformation or an acquired lesion as a result of choroidal atrophy or scarring following choroiditis [5]. 

Morphologically, there are two FCE types—without separation between the photoreceptors and the retinal pigment epithelium (RPE) (conforming FCE, type 1), and when the photoreceptors appear to be detached from the underlying RPE with a hyporeflective subretinal space (non-conforming FCE, type 2) [3]. Choroidal excavation has an unusual clinical appearance, visible on fundoscopy as RPE abnormalities or a round or fusiform yellowish lesion [1,2,3]. 

FCE may be an isolated lesion or associated with other ocular conditions [5,6,7,8,9,10,11,12,13,14,15]. The excavation can occur in the same eye with other pachychoroid disease or in the fellow eye. According to Ellabban et al., the prevalence of FCE in 116 eyes with Central Serous Chorioretinopathy (CSC) was 7.8% [8]. Another study showed that 4.6% of 243 eyes with neovascular Age-Related Macular Degeneration (nAMD) or Polypoidal Vasculopathy (PCV) presented similar morphological lesions in the choroidal tissue [9]. Associations of FCE with macular neovascularization have also been found, although most FCE cases remain stable over several years of follow–up [3,5]. Lee et al. reported both type 1 and type 2 MNVs in their 16 Korean patients with FCE [16]. The authors hypothesized that impaired choroidal circulation and hypoperfusion within the FCE may be the cause of secondary MNV. 

Precise epidemiological data on FCE are still unknown. It is already known that FCE is more common in the Asian population, although the significance of these associations remains unclear. In the available studies, FCE was reported more frequently in women than in men [7,16].

Multimodal imaging is recommended for the detection and differential diagnosis of pachychoroid spectrum diseases, and OCT is considered particularly important as it allows detailed visualization of the retinal structure [5,7]. Swept source OCT (SS–OCT) uses a longer wavelength than SD–OCT (1050 nm versus 840 nm) and enables better visualization of the choroid [17]. OCT angiography (OCTA) is a novel technique for non–invasive imaging of the retinal and choroidal vessels, currently widely used in the diagnosis of MNV [18,19]. 

The purpose of the study is to present the epidemiology, clinical features and multimodal imaging findings in our patients with FCE.

## 2. Materials and Methods

This is a case series of consecutive patients with a diagnosis of FCE, confirmed by multimodal imaging, from a review of 5076 optical coherence tomography scans in 2538 patients. They attended our outpatient clinic from January 2019 till November 2022. The study was carried out in accordance with the tenets of the Declaration of Helsinki. 

All patients underwent a complete ophthalmic examination, including slit lamp biomicroscopy with dilated fundus examination, best-corrected visual acuity (BCVA), refractive error measurement and intraocular pressure (IOP), using an air puff tonometer (Canon TX-20 P, Canon Medical Systems Europe). Multimodal imaging was performed using SS-OCT (DRI OCT Triton; Topcon, Tokyo, Japan) and included color fundus photography, fundus autofluorescence (FAF), OCT, en-face OCT and OCT angiography. The 9 mm radial OCT B-scan centered on the fovea was performed to obtain high-quality images of the retina and choroid. FCE was defined as a localized choroidal depression confirmed by OCT, without concomitant scleral ectasia or posterior staphyloma and other conditions with choroidal thinning. CT was measured manually using built-in calipers in the OCT software and was defined as the distance between the hyperreflective line corresponding to the outer boundary of the RPE and the hyperreflective line corresponding to the chorioscleral border. The measurements were obtained in the subfoveal region and in the area of the maximum choroidal thickness in the affected eye and under the fovea in the fellow eye. The diagnosis of concurrent retinal pathologies was based on clinical examination and OCT. MNV was confirmed using OCTA. OCTA was performed using 3 mm × 3 mm images of the macula. The OCTA system automatically segments the area into four layers: superficial capillary plexus, deep capillary plexus, outer retina layer and choriocapillaris. In the case of automatic segmentation errors, the boundaries of the slab were corrected manually. Low-quality scans with motion artifacts or blurred images were excluded from the final analysis. 

Patients were recommended to have follow-up visits every six months and to perform self-monitoring once a month using the Amsler test. 

Study data were analyzed with the Statistical Package for Social Sciences (SPSS) for Windows version 22.0 (SPSS Inc., Chicago, IL, USA). The descriptive statistics were shown as mean values ± standard deviation.

## 3. Results

In this retrospective study, 14 consecutive patients were enrolled: 13 female and 1 male. The mean age of the subjects was 40 ± 13.58 years (median (Me) = 36.5; range: 23–64 years). All of them were Caucasian in good general health. There was no history of pachychoroid spectrum diseases in patients prior to excavation diagnosis. Eight eyes were myopic (57.14%), four emmetropic (28.57%) and two hypermetropic (14.28%); the refractive error ranged from −4.5 diopters (D) to 3.00 D. The BCVA in our study group ranged from 0.7 to 1.0. In all patients, IOP was within normal limits (ranging from 12 mmHg to 20 mmHg), and there were no significant differences in IOP between the eye with FCE and the healthy eye. Patients’ demographic and clinical characteristics are summarized in Table 1.

**Figure 1:**
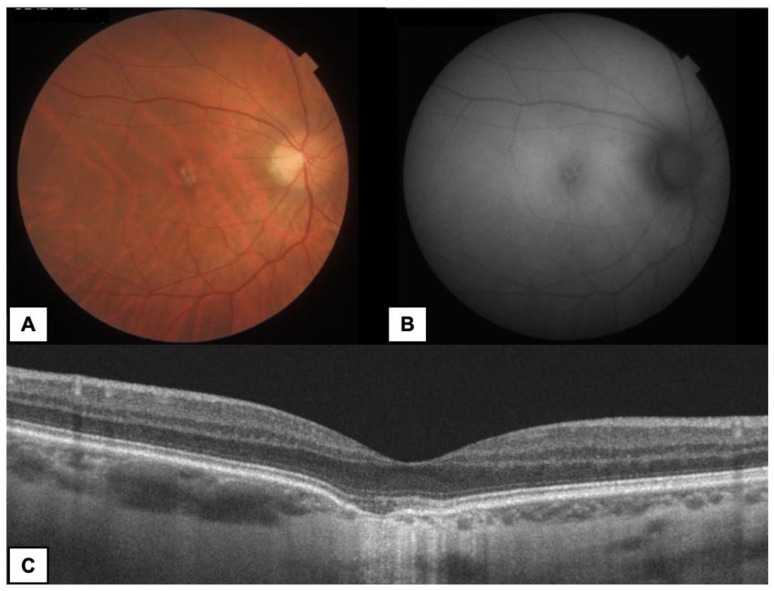
A 35-year-old woman with subfoveal FCE type 1 in the right eye (RE). (**A**) Color fundus photography—yellowish lesion in the center of macula. (**B**) FAF—irregular hypoautofluorescence in the area of FCE. (**C**) OCT B-scan—conforming FCE with RPE atrophy and choroidal thinning at the base of FCE.

**Figure 2:**
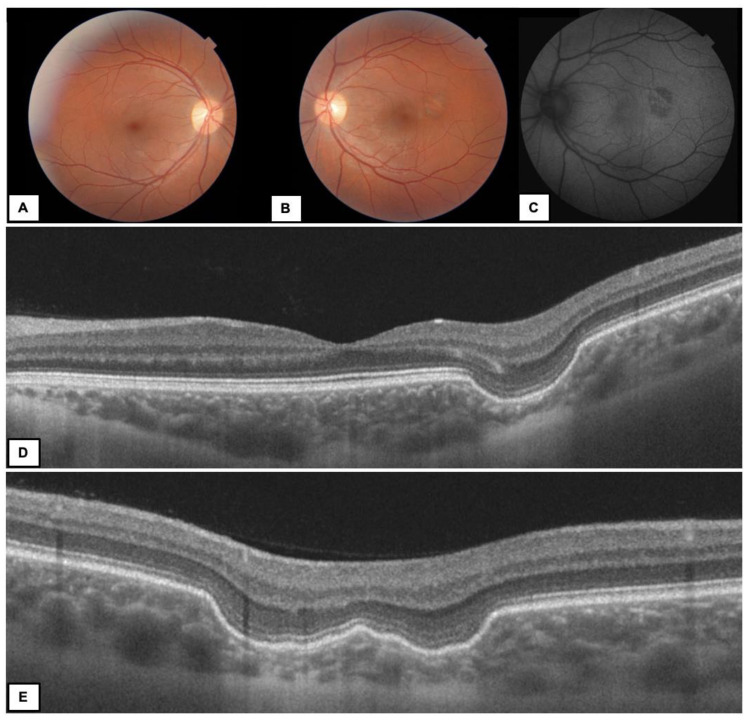
A 37-year-old woman with FCE type 1 in the left eye (LE). Color fundus photography (**A**) RE—normal fundus (**B**) LE—yellow–grey round area of FCE located juxtafoveally (**C**) FAF—irregular hypoautofluorescence in the area of FCE corresponding to the RPE changes (**D**,**E**) OCT B-scans—different projections of the choroidal ectasia on radial scans (double-bowl-shaped FCE).

**Figure 3:**
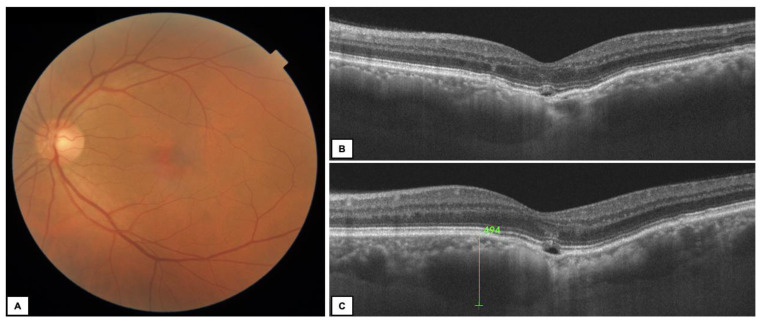
A 64-year-old man with subfoveal FCE type 2 in the left eye. (**A**) Color fundus photography—subtle alteration of the RPE in the fovea; (**B**,**C**) OCT B-scans—non-conforming FCE with increased CT (494 μm) and pachyvessels nasally to the lesion (**C**).

FCE occurred unilaterally and was an isolated lesion in all cases. The fellow eyes did not show any macular pathology in the entire group. Twelve eyes (85.7%) presented type 1 FCE (Figure 1 and Figure 2), while type 2 FCE was revealed in two eyes (14.3%) (Figure 3).

Various forms of ectasia were observed in the study group. Figure 2E shows OCT B-scans of the mixed shaped (double-bowl-shaped) FCE.

**Figure 4:**
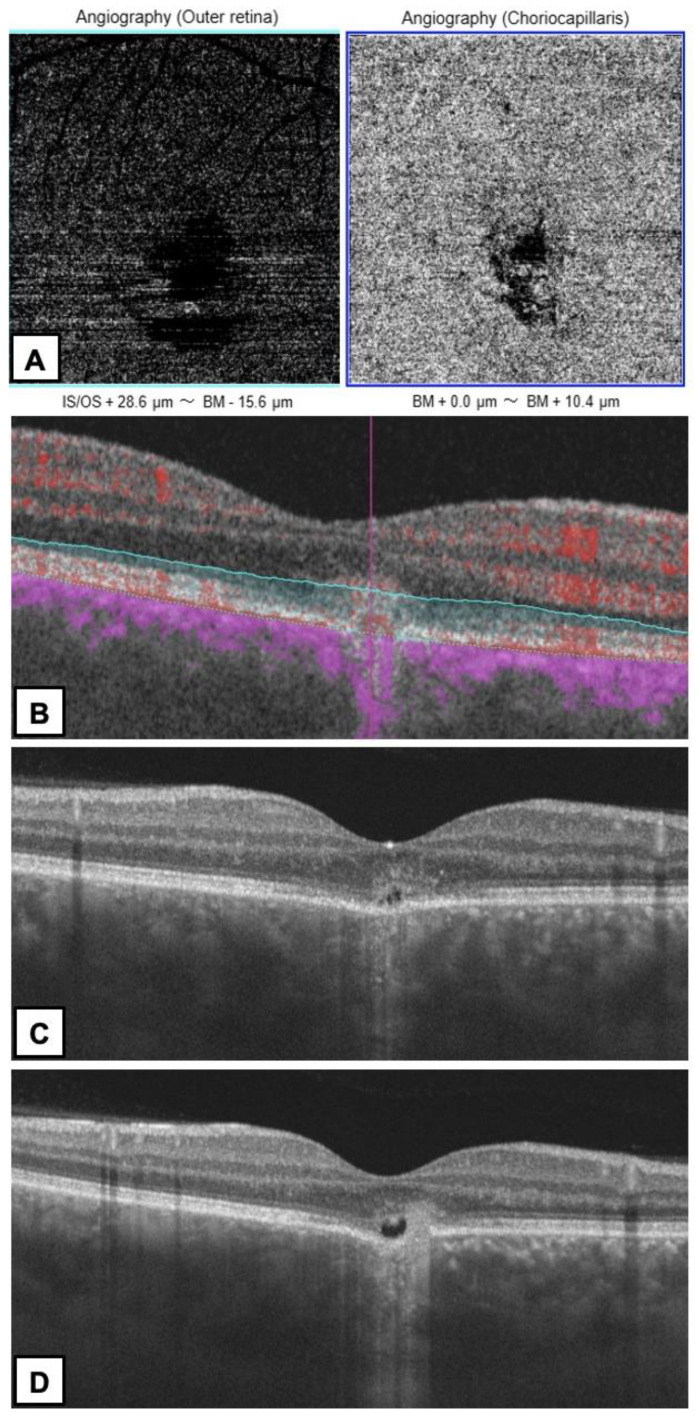
A 27-year-old woman with MNV complicating FCE type 2 in the right eye. (**A**) OCTA—small neovascular network visible in the outer retina and choriocapillaris layers with an adjacent dark halo. (**B**) The corresponding B-scan with adjusted segmentation boundaries and flow overlay reveals flow signal in the MNV (**C**,**D**) OCT B-scans—non-conforming FCE with disruption of the outer retinal layers by MNV.

In 79% of cases, the FCE was subfoveal, while others were located outside the fovea (juxta- or extrafoveally). Subfoveal CT varied from 55 to 285 μm (mean 140.36 ± 70.02 μm, Me = 129 μm) in the affected eye, depending on the location of the FCE—the choroid was the thinnest at the base of the FCE. The maximum CT was observed in the area adjacent to the FCE and ranged from 330 to 517 μm (mean 389.86 ± 54.84 μm, Me = 379 μm), with the presence of pachyvessels. Thirteen patients had normal CT and no presence of pachychoroid in their fellow eye. In the healthy eye, the maximum CT was observed in the subfoveal area and ranged from 246 to 518 μm (mean 288.07 ± 68.69 μm, Me = 269.5 μm). We observed pachychoroid features in the fellow eye only in a patient with neovascularization (CT = 518 μm). OCTA confirmed MNV complicating non–conforming FCE in one eye in a 27-year-old woman (Figure 4).

This eye was treated with intravitreal antivascular endothelial growth factor (anti-VEGF)—three doses of aflibercept—with poor visual response; the patient is now under observation. 

Only one patient reported blurred vision and metamorphopsias in the affected eye (with secondary MNV). The remaining 13 individuals were asymptomatic; FCE was an accidental finding during a routine ophthalmological examination. 

The characteristics of FCE in our patients, including type, location, maximum CT, contralateral eye status and presence of MNV are summarized in Table 2.

The mean follow–up time in our study group was 16 ± 12 months with Me = 12.5 months.

## 4. Discussion

The etiopathology of FCE remains a topic of active research and is still unknown. It has been suggested that this clinical entity may be a morphological manifestation of multiple processes and may be congenital or secondarily acquired [6]. 

The epidemiology of FCE is also uncertain. In one study, FCE was found in 0.18% of eyes in patients <40 years of age [11]. In this study, FCE was found in 14 Caucasian patients among the 5076 SS–OCT scans (2538 patients). In comparison, Chung et al. found 16 Chinese FCE patients when screening 4436 OCT scans [5]. Our patients were aged 23–64, similar to the group described by Margolis [3]. As we do not have precise epidemiological data on this disease (but it is classified as rare), our series of 14 cases adds important information to the available knowledge on FCE. Recent studies have shown that choroidal ectasia is more common in the Asian population; however, the significance of this association remains unclear [10]. All our patients were Caucasian, which is not very typical for FCE and made this group unique. The disease has been reported much more frequently in women than in men [7], which we confirmed in our study group (13 women, 1 man). Most of the previously described FCE patients were myopic [2,3], which is consistent with our findings (8 of 14 patients had associated moderate myopia), and this may confirm that myopia is one of the main risk factors for FCE. 

FCE may accompany other diseases or may be an isolated lesion. Some cases of FCE have been reported in patients with many different pathologies such as pachychoroid spectrum diseases, AMD, bestrophinopathies, Epstein-Barr Virus (EBV) infection and Multiple Evanescent White Dot Syndrome (MEWDS) [8,9,10,11,12,13,14,15]. Therefore, the OCT images of FCE in patients with concomitant CSC or PCV may show: serous retinal detachment; serous retinal pigment epithelium detachment (PED); serous, hemorrhagic, multiple PEDs; elongation of photoreceptor outer segments; retinal fluid; as well as RPE abnormalities, including the type of clusters and irregularities resembling soft drusen (pachydrusen) [7]. Contrary to the studies cited above, we did not reveal any other ocular pathology in either the affected or the fellow eye, with the exception of one case in which FCE was complicated by MNV. This neovascular complex was located within the boundary of excavation, similar to most of the patients in Lee’s study [16]. The author of that study suggested that this close topographic relationship confirms FCE’s involvement in the formation of MNV. In addition, mechanical stretching of the RPE/Bruch’s membrane may result in a focal break in the Bruch membrane and induce neovascularization. Margolis et al. also found one case with MNV in their group, but in this eye FCE coexisted with CSC [3]. It is worth noting that we confirmed choroidal thickening in the patient’s fellow eye and that she had the thickest choroid in the affected eye in the group. This may suggest that the thicker the choroid, the higher the risk of neovascularization; however, one case is not enough to draw such conclusions. In the remaining cases, the CT of the fellow eye was normal. In contrast to our results, Inanc et al. revealed no statistically significant difference in the CT between the area adjacent to the FCE and CT in the fellow eye in a group of 13 patients [6]. We found pachychoroid features in the affected eye in the whole group, which has confirmed that FCE is strongly associated with thickened choroid. 

Our CT measurements showed that the choroid was the thinnest at the base of the FCE, and the results are consistent with the findings by Inanc et al. [6]. In both healthy eyes and most eyes with pachychoroid (CSC, PCV), the maximum CT is typically located in the subfoveal region and the choroid is the thinnest in the nasal part of the macula [4,20]. In Peripapillary Pachychoroid Syndrome (PPS), the thickest choroid was described nasally [20]. In contrast to other pachychoroid spectrum diseases, in the cases of FCE, the maximum CT can be found in different regions—it depends on the location of the FCE and usually is observed in the area adjacent to FCE. Therefore, CT measurement in the area of maximum choroidal thickness seems to be a more valuable parameter than measurements in the fovea, where most of the FCEs are located.

In all our patients, FCE occurred unilaterally; however, single bilateral cases have been reported in the literature [3,21]. More than one area of FCE has been described in some eyes [3]; we did not confirm these findings in our study group. In previous reports, choroidal ectasia has been classified by location as foveal or extrafoveal. Foveal location was divided into subfoveal or juxtafoveal, which are the most common types. Extrafoveal lesions have also been reported [22]. In our study, most of the lesions were localized under the fovea, which is similar to the available literature [3]. We have observed only one case with extrafoveal choroidal ectasia.

Patients with extrafoveal FCE usually do not report any clinical symptoms, which are usually related to subfoveal FCE and include central scotoma, metamorphopsias and deterioration of visual acuity [21]. Metamorphopsias were noted in all three patients described by Wakabayashi et al. [2]. In contrast to the aforementioned study, only one of our patients reported visual disturbances in the affected eye, consisting of blurred vision and reduced visual acuity (patient with complicating FCE (FCE-MNV)). Others, despite FCE’s location in the foveal area, reported no complaints. During the follow-up, the course of the disease in the study group was stable, which is consistent with Chung et al. [7]. 

In the previous studies, two types of choroidal excavation were described [7]. Twelve of our patients presented conforming FCE (type 1). Type 2 FCE was found in 14.3% of our study group (2 eyes), similar to Chung et al.’s report [5]. Shinojima et al. divided the choroidal ectasia into cone-shaped, bowl-shaped and mixed morphology, according to the shape of the choroidal concavity itself [23]. Our patients presented all types of the shapes, but the most interesting was the mixed type (double-bowl-shaped) FCE. This case showed that radial OCT B-scans are very useful in the diagnosis of FCE, as they show the lesion in different projections, and we can be sure what the shape of the excavation really looks like.

The differential diagnosis for FCE includes posterior staphyloma, conditions with choroidal thinning, uveal tumors, vitreomacular traction, impending macular hole, macular pseudohole, CSC, myopic schisis, soft drusen and several congenital defects [21]. Multimodal imaging allows us to differentiate pathological changes and to make the right diagnosis.

Many investigators have proven the invaluable role of multimodal imaging in pachychoroid spectrum diseases [1,5,6,7,20]. There are many methods used in FCE diagnostics, including, e.g., color fundus photography, fundus autofluorescence, fluorescein angiography (FA), indocyanine angiography (ICGA), OCT and OCTA [22,23,24,25,26,27,28,29]. Based on our case series, we revealed that the fundus lesions in FCE were uncharacteristic and presented atypical pigmentary changes, so multimodal imaging was required to confirm the correct diagnosis. All affected eyes manifested irregular hypoautofluorescence on FAF images, similar to the observations of Margolis et al. [3]. These authors also found hypoautofluorescence with areas of hyperautofluorescence on FAF images from one patient with FCE and a history of resolved CSC. We agree with the results of previous studies that, among other imaging modalities, OCT B-scans provide the most important information about FCE morphology and features. Additionally, the swept-source OCT used in the study allowed us to visualize the choroid better than SD–OCT.

Contrary to other authors [2,3,20,23], we did not perform invasive diagnostic methods (FA and ICGA) in our patients. Various reports confirmed that FA was unremarkable in eyes with FCE, showing varying degrees of hyperfluorescence, consistent with transmission defects, or hypofluorescence, when the RPE was intact [2,3]. ICGA showed mainly hypofluorescence correlating with impaired choroidal circulation and hyperpermeability in some cases [23]. In our opinion, there was no indication to perform additional invasive tests in the study group. In the diagnostic process, we relied on OCTA, which allowed us to confirm MNV in one eye. According to the recent literature, OCTA is a good non-invasive, alternative imaging technique for the diagnosis of MNV and should be considered as a useful imaging tool in pachychoroid spectrum diseases [18,19,27,28,29]. Demirel et al. concluded that OCTA seems to be a superior technique to ICGA with regard to revealing MNV over the areas that show pachychoroid characteristics [27].

The limitations of the study are the small group of patients and its retrospective nature. Further studies are needed to expand the available knowledge about FCE etiology and clinical course.

## 5. Conclusions

Our study confirms that FCE is a rare ocular condition, but it may be more common in the Caucasian population than previously thought. The prevalence of FCE is probably underestimated due to its asymptomatic nature and uncharacteristic fundus presentation. Multimodal imaging methods, mainly OCT, are crucial in FCE diagnosis and monitoring. As with other pachychoroid spectrum diseases, FCE can be complicated by macular neovascularization, and non-invasive OCTA should be performed to confirm the presence of MNV and to initiate treatment with anti-VEGF therapy.

## Figures and Tables

**Table 1:** Demographic and clinical characteristics of patients with FCE.

Characteristics	Patients (*N* = 14)
Age (range) [years]	23–64
Sex, No. [%]	13 female (93%), 1 male (7%)
Race, No. [%]	14 Caucasian (100%)
BCVA (range)	0.7–1.0
Refractive error [D] (range)	(−4.5)–(3.0)
IOP (mmHg)	12–20

**Table 2:** FCE characteristics and fellow eye condition in the study group.

Patient (Age in Years)	FCE Type (1 or 2)	FCE Localization	Maximum CT in the Affected Eye [μm]	Maximum CT in the Fellow Eye [μm] (Subfoveal CT)	Second Eye Condition	MNV Presence
1 (28)	1	Subfoveal	365	255	Without abnormalities	NO
2 (50)	1	Subfoveal	341	246	Without abnormalities	NO
3 (23)	1	Juxtafoveal	420	263	Without abnormalities	NO
4 (64)	1	Subfoveal	382	258	Without abnormalities	NO
5 (48)	1	Juxtafoveal	376	275	Without abnormalities	NO
6 (51)	1	Subfoveal	330	248	Without abnormalities	NO
7 (26)	1	Subfoveal	395	295	Without abnormalities	NO
8 (30)	1	Subfoveal	345	252	Without abnormalities	NO
9 (35)	1	Subfoveal	360	278	Without abnormalities	NO
10 (38)	1	Subfoveal	385	283	Without abnormalities	NO
11 (35)	1	Extrafoveal	353	264	Without abnormalities	NO
12 (43)	1	Subfoveal	395	298	Without abnormalities	NO
13 (64)	2	Subfoveal	494	300	Without abnormalities	NO
14 (27)	2	Subfoveal	517	518	Increased choroidal thickness (518 μm)	YES

## Data Availability

Data are available from the corresponding author upon reasonable request.
